# A Case of Proximal Humerus Fracture Metastasis due to Clear Cell Renal Carcinoma Treated With Modular Hemiarthroplasty

**DOI:** 10.1155/cro/2988571

**Published:** 2026-09-29

**Authors:** Gianfranco Puddu, Marco Piras, Stefano Congia

**Affiliations:** ^1^ Orthopaedic and Traumatology Division, Nostra Signora di Bonaria Hospital, ASL Medio Campidano, San Gavino Monrele, Italy

## Abstract

We present a clinical case of a patient with proximal humeral bone metastasis from renal carcinoma. The patient underwent bone metastatic lesion resection, followed by shoulder modular hemiarthroplasty using a revision stem and an extended articular surface humeral head. The patient survived 3 years, maintaining sufficient shoulder function for activities of daily living and remaining pain‐free (Constant Shoulder score 54 points and DASH score 29.3 at 2 years follow‐up). The use of hemiarthroplasty proved to be an effective treatment option for this patient, considering the functional requirements and life expectancy.

## 1. Introduction

Bone metastases are a common complication in patients with clear cell renal carcinoma (CCRC) [1], one of the most common and aggressive forms of kidney cancer.

CCRC accounts for about 2%–3% of all malignant tumors in adults and has a predilection for metastasizing to the bones, lungs, liver, and brain.

Bone metastases occur in about 30% of patients with advanced renal carcinoma and can cause significant complications, including severe pain, pathological fractures, and spinal compression.

Fractures from bone metastases represent a complex clinical challenge and require a multidisciplinary approach for optimal management [2].

These fractures are often the result of a combination of osteolysis induced by tumor proliferation and the alteration of bone microarchitecture, which makes the affected bones extremely fragile.

The treatment of pathological fractures caused by bone metastases includes a combination of surgical interventions, radiotherapy, and systemic therapies, aimed not only at stabilizing the bone and relieving pain, but also at controlling the progression of the cancer. In the context of CCRC, the management of pathological fractures becomes even more critical due to the aggressive nature of the tumor and its variable response to conventional treatments.

The therapeutic approach must be personalized, considering the patient′s overall conditions, the extent of metastatic disease, and the location of the bone lesions. This article presents a clinical case of proximal humeral pathological fracture in a patient with CCRC.

## 2. Case Report

We present the case of a 54‐year‐old male patient with a history of opioid dependency receiving pharmacological treatment (Section 4.1).

In December 2022, he sustained low‐energy trauma at home, followed by worsening pain in the subsequent days, leading him to visit the provincial hospital.

Radiographic examination revealed a fracture of the surgical neck of the humerus extending to the proximal diaphysis (Figure 1). Given the nature of the trauma and radiographic findings, a sling brace was placed for pain relief, and a CT scan of the upper limb was performed, showing a significant osteolytic area in the humeral head and the proximal diaphysis.

**Figure 1:**
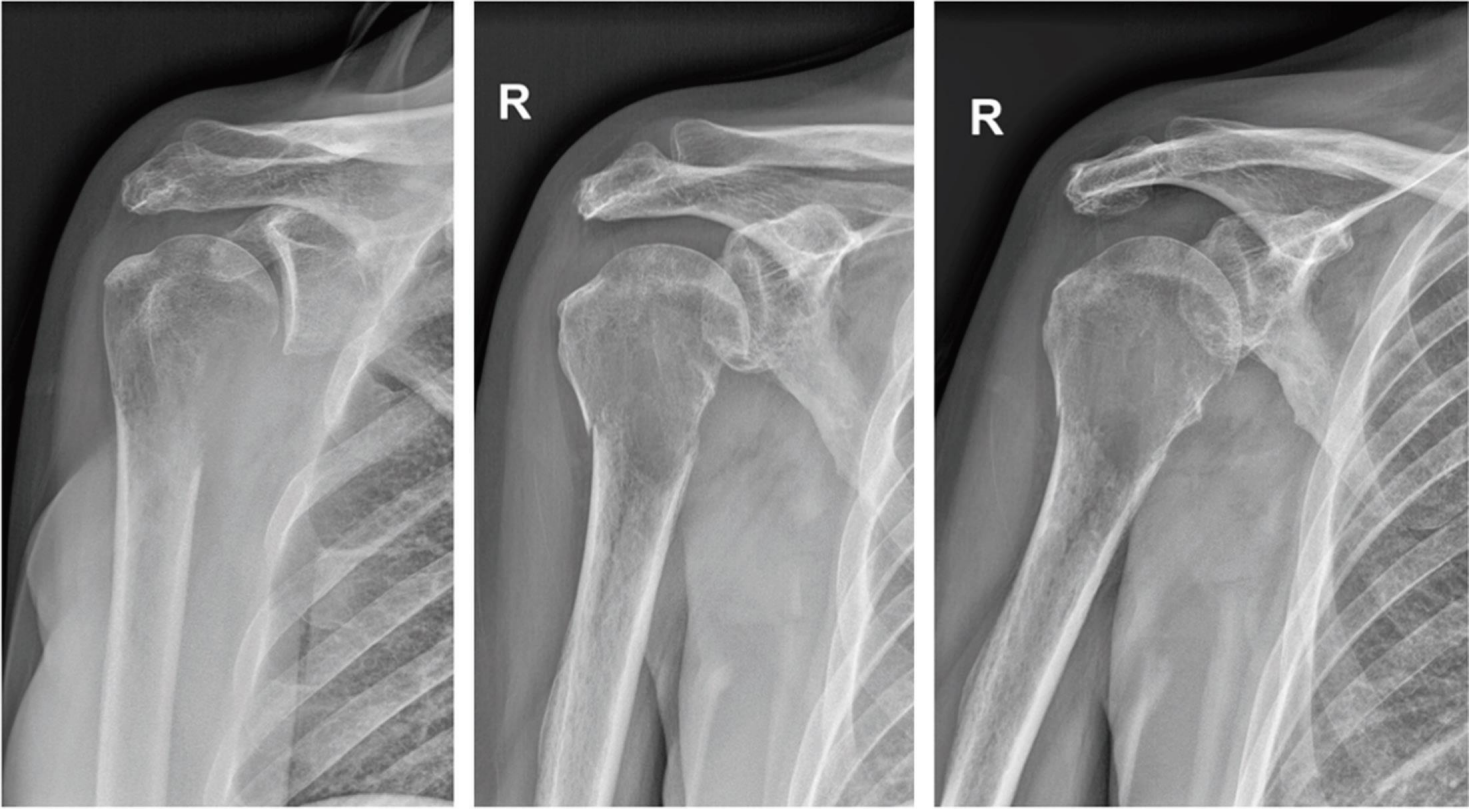
X‐rays performed upon admission in the emergency department.

The presence of tissue invading the subscapular region and the articular side of the shoulder was reported.

An oncological consultation was conducted, followed by a total body CT scan, which revealed questionable nodulations for metastases at the pulmonary level and a coarse formation at the left kidney.

According to the oncologist, the tumor was classified as Stage IV based on the TNM classification system [3].

Following further MRI, which confirmed the total disruption of the shoulder tissues due to the proliferating tissue, the fracture was reported as a pathological fracture secondary to metastasis from renal neoplasia (Figure 2).

**Figure 2:**
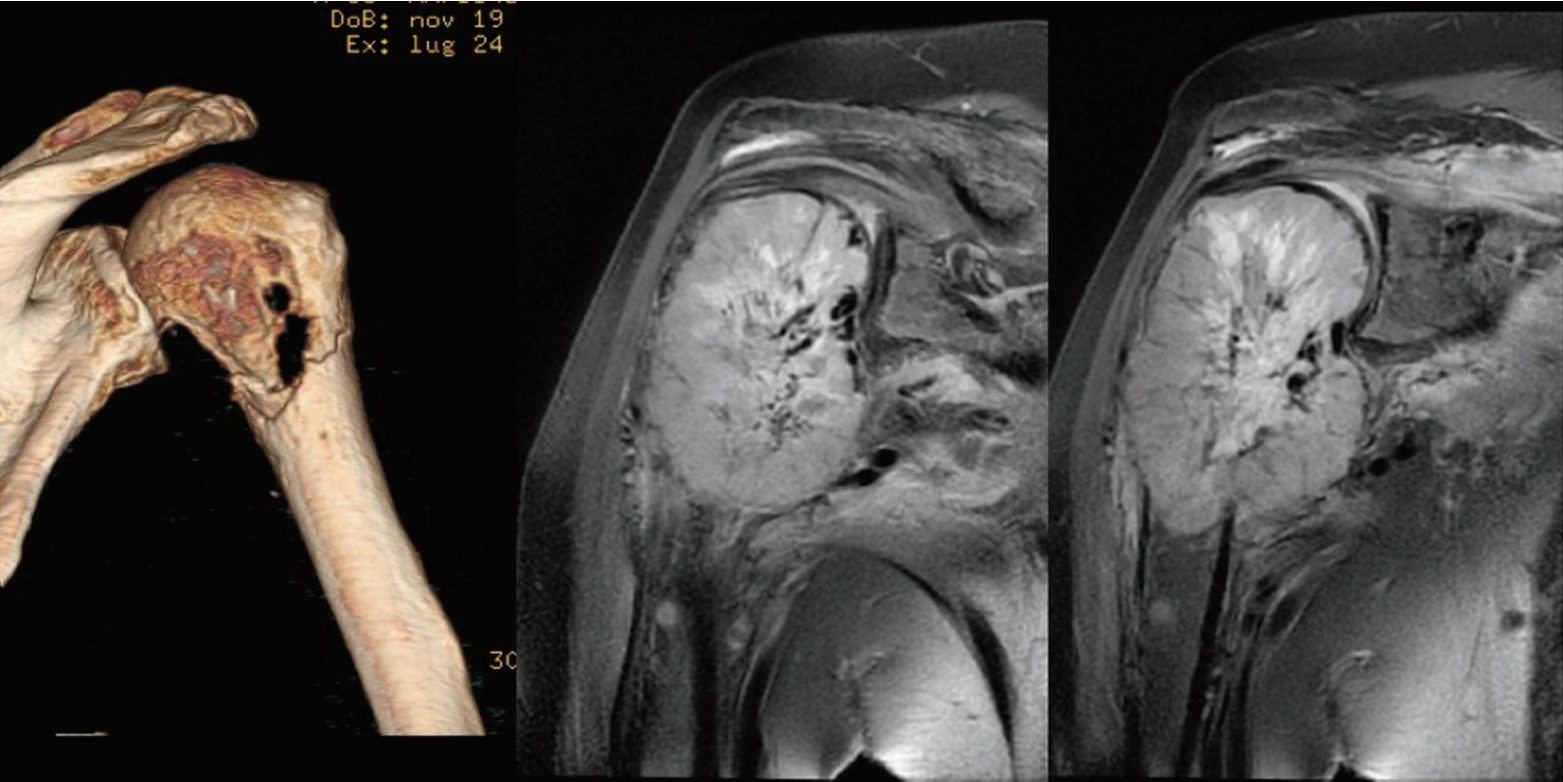
CT and MRI of proximal humerus fracture.

A multidisciplinary consultation with oncologists and anesthetists was held, and the patient was referred to a national reference center for orthopedic oncological pathologies, but he refused.

In February 2023, surgical removal of the mass and implantation of a megaprosthesis for resection at the right shoulder were performed for pain relief and to ensure functional autonomy in daily life. The removed tissue was sent to pathology for analysis and typing of the neoplasm for staging and setting up oncological therapy.

## 3. Surgical Technique

The patient was placed in a beach chair position, and 2 g of cefazolin IV was administered as preoperative prophylaxis. Throught an extended deltopectoral approach, a gray‐brownish tissue infiltrating the soft tissues and largely replacing the proximal humerus was found.

The neoplastic bone tissue—including the humeral head, which showed total subchondral bone disruption with only the cartilaginous structure remaining—was removed, and the sample was prepared for sending to the department of pathology (Figure 3).

**Figure 3:**
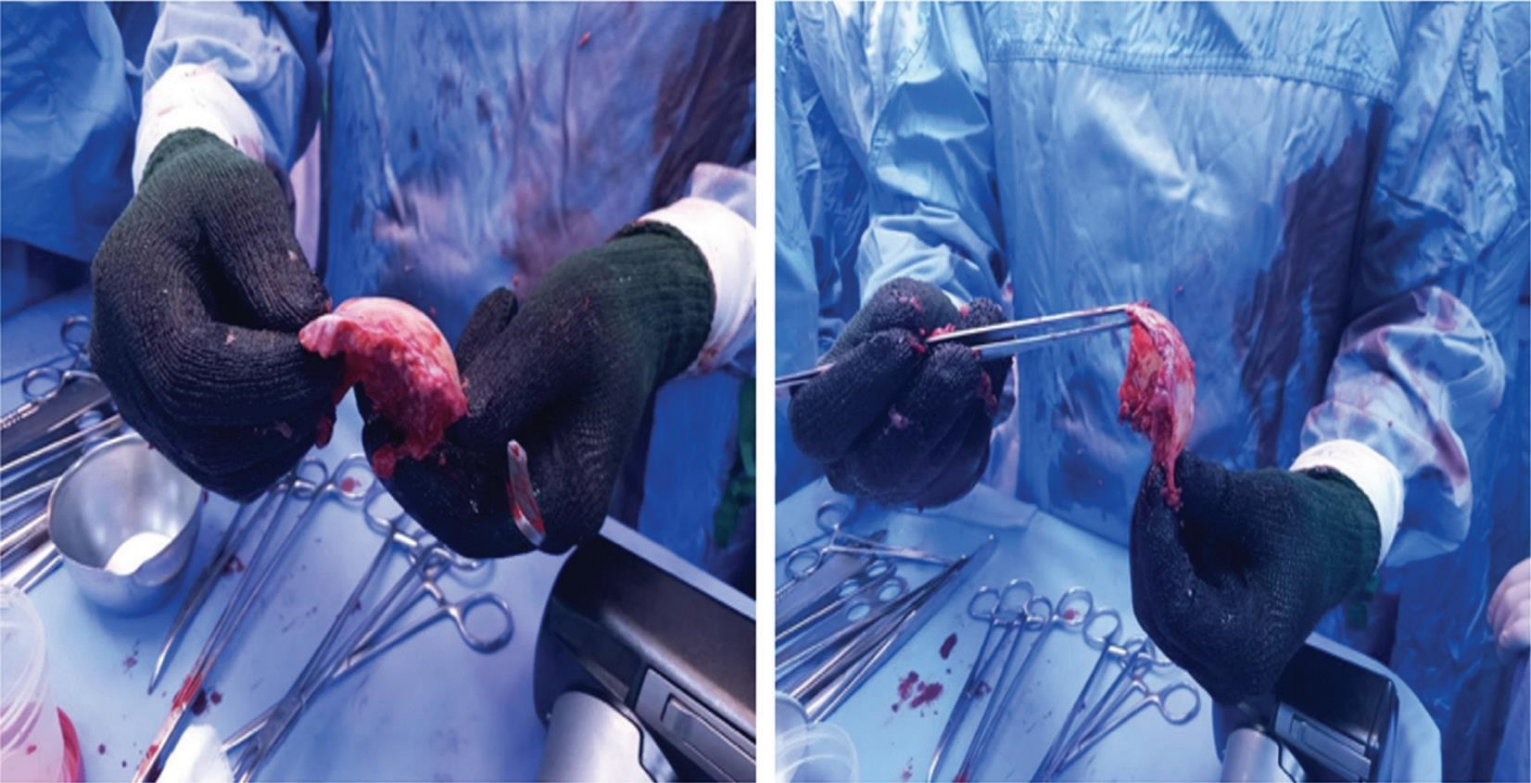
Intraoperative view of the cartilage and subchondral residue of the humeral head.

Using increasingly sized rasps, the healthy bone was milled, and a modular Comprehensive SRS Zimmer Biomet endoprosthesis was implanted (Figure 4).

**Figure 4:**
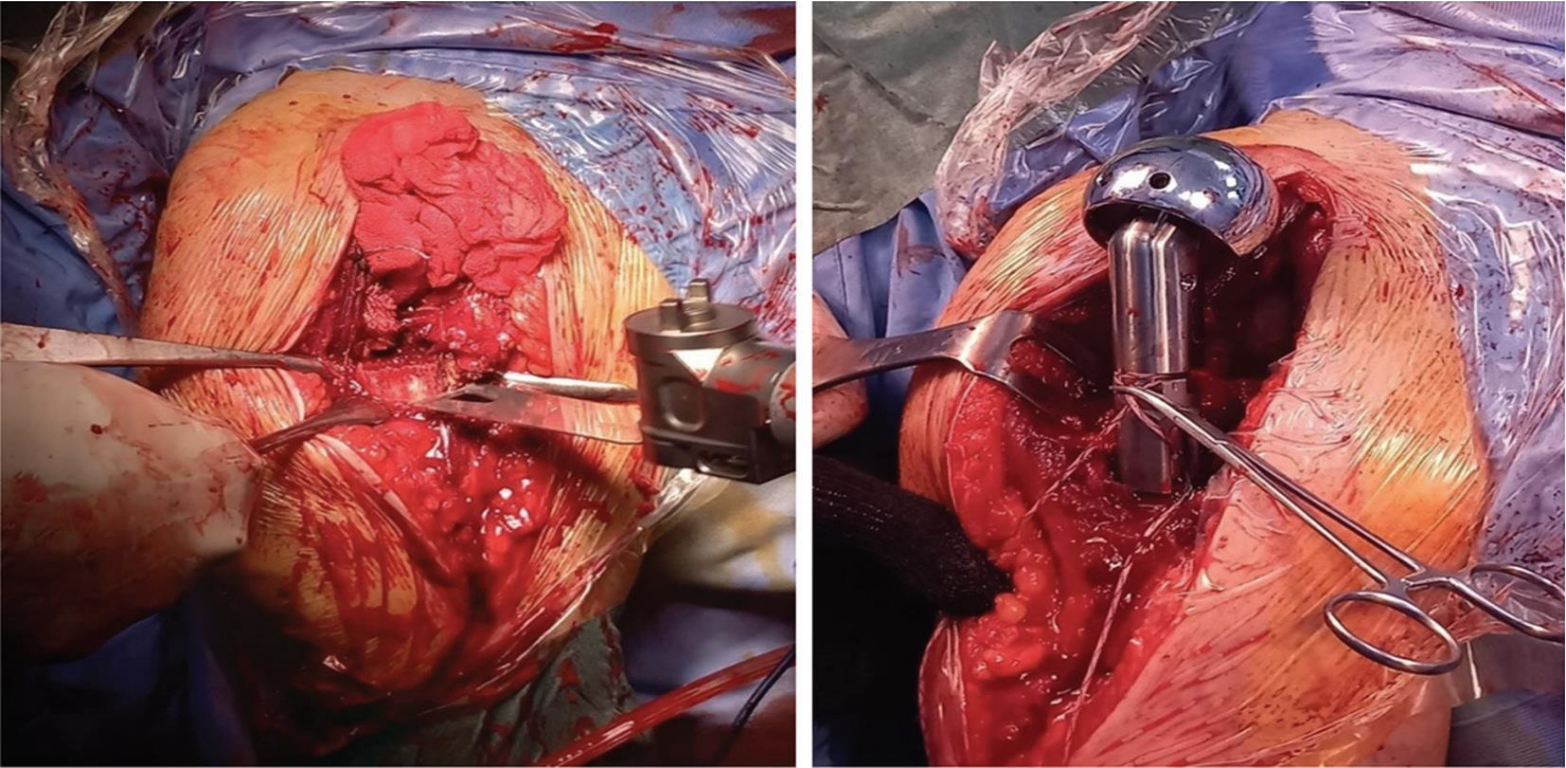
Intraoperative humeral resection and placement of trial implant.

We used a cemented humeral stem 150 mm long with a diameter of 9 mm, an intercalary segment of 30 mm, and a proximal humeral body of 58 mm. The humeral head was a super extended articular surface (EAS) 17 mm long with a diameter of 44 mm.

We fixed part of the the healthy tendinous structures of the rotator cuff to the prosthetic stem.

## 4. Results

During the postoperative course, the patient experienced anemia and received a transfusion of one unit of packed red blood cells.

The patient had a preoperative hemoglobin level of 11.4 g/dL. Given the hypervascular nature of the metastatic lesion [4], three units of packed red blood cells were reserved preoperatively. One unit was transfused on the first postoperative day when the hemoglobin level was 7.9 g/dL. The patient was discharged with a hemoglobin level of 9.7 g/dL.

The postoperative check showed the prosthesis was well‐positioned and articulated with the glenoid (Figure 5). The morphopathological examination revealed the presence of proliferative tissue suggestive of CCRC. Further family history revealed a genetic basis for the disease (father and brother affected by the same pathology). A follow‐up oncological plan for metastatic renal carcinoma was established, and oncological therapy with pembrolizumab and axitinib was started [5].

**Figure 5:**
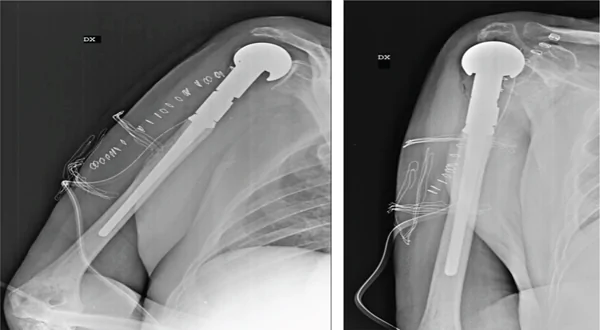
X‐ray postoperative.

At the 1‐month follow‐up, mild scapular plane mobility was noted.

The radiographic control remained unchanged; the patient was subjectively satisfied and reported no pain in the operated shoulder.

In February 2024 (1 year of follow‐up), the patient reported a subjective improvement in mobility with active anterior elevation of 70° and no shoulder pain; the x‐ray was unchanged compared with the postoperative follow‐up.

The total Constant Shoulder score was 49 points, and the DASH score (Disability of the Arm, Shoulder, and Hand Score) was 33.6, showing improvement in ROM in flexion‐extension and in intrarotation and extrarotation. The patient was independent in activities of daily living (ADL).

In February 2025 (2 years of follow‐up), the patient reported no pain, had no limitations in ADL, scored 54 points according to the Constant score, and scored 29.3 according to the DASH (Figure 6). The x‐ray showed no loosening of the implant and an upward migration of the humeral head with “acetabularization” (Figure 7).

**Figure 6:**
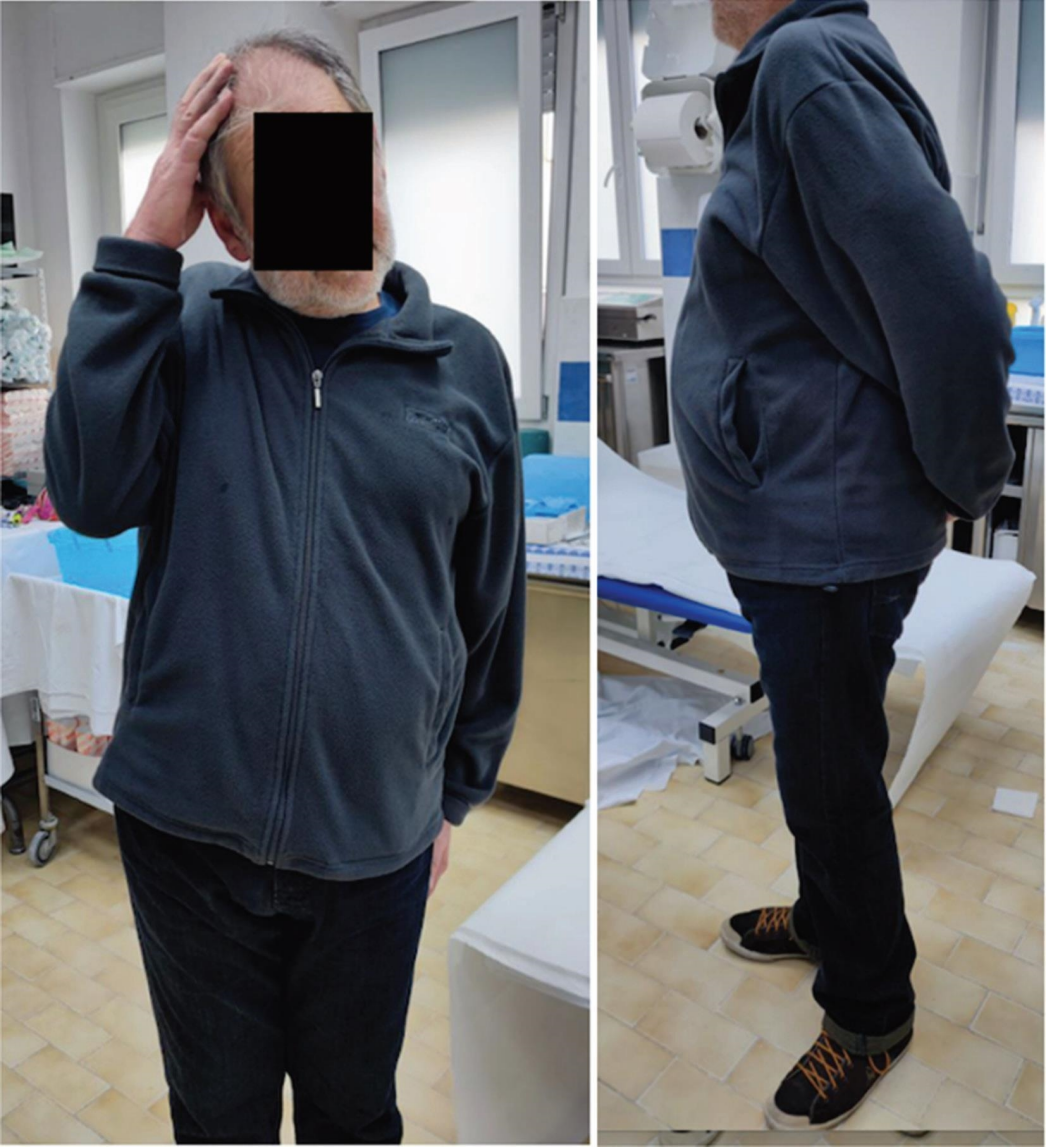
The patient while pretending to comb his hair and take the wallet from his trouser pocket after 2 years.

**Figure 7:**
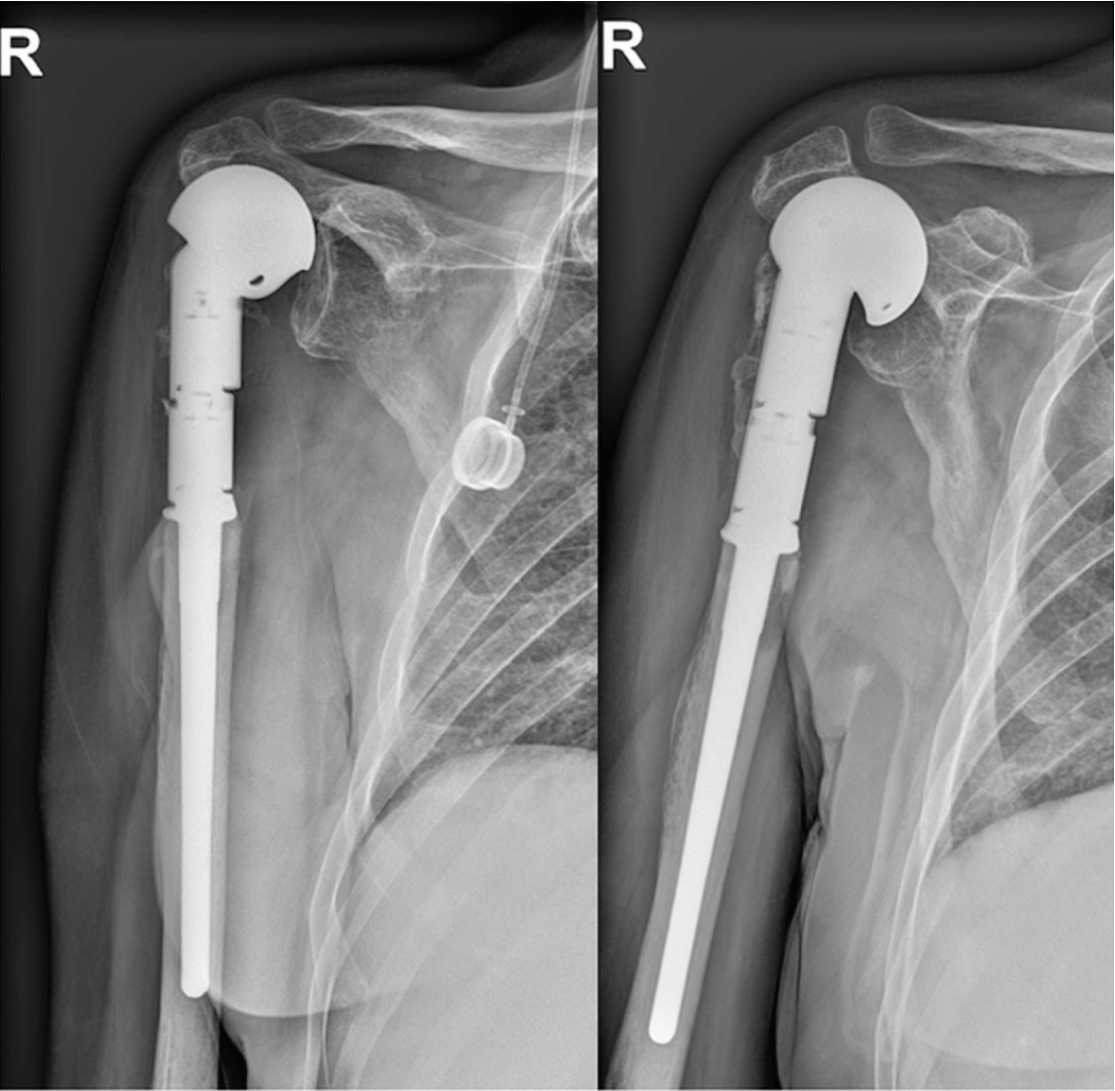
X‐ray after 2 years.

At the 1‐ and 2‐year follow‐ups, the patient expressed satisfaction with the procedure and good pain control, despite residual functional limitations that remained compatible with an acceptable quality of life.

In December 2025, the patient was contacted to schedule a clinical and radiographic follow‐up for February 2026. However, due to a decline in his general health (suspected pneumonia of unspecified nature), he declined the planned evaluation. He agreed to complete the DASH questionnaire by telephone, which yielded a score of 46.2.

In February 2026, the patient died at home; the exact circumstances were not reported.

### 4.1. Timeline Summary

• December 2022: a 54‐year‐old male patient sustained fracture of the right humerus after low‐energy trauma.
• January 2023: diagnosis of a pathological humeral fracture, Stage IV left kidney CCRC, and a multidisciplinary consultation.
• February 2023: surgical removal and implantation of a right shoulder modular hemiarthroplasty, followed by oncological therapy.
• February 2024: 1‐year follow‐up.
• February 2025: 2‐year follow‐up.
• December 2025: patient refused to schedule clinical and radiographic follow‐up due to deterioration of his general condition.
• February 2026: death of the patient.

## 5. Discussion

Pathological fractures due to bone metastases represent a significant challenge in the management of oncological patients, especially when the primary tumor is as aggressive as in the case of CCRC [6]. The management of these fractures requires a multidisciplinary approach that integrates oncological, orthopedic, and anesthesiological expertise to ensure optimal and personalized treatment [7].

It has been reported that, in some instances, the first clinical manifestation of CCRC may present as metastasis to the proximal humerus, initially causing pain and subsequently resulting in a pathological fracture [8]. Bianco Prevot et al. [7] described a case of proximal humeral metastasis from renal carcinoma in the absence of an identifiable primary lesion. In their report, a metastasis of unknown primary was diagnosed following the failure of intramedullary nailing used to treat a proximal humerus fracture. Despite the inability to identify the primary tumor, the patient was managed as a case of bone metastasis from renal carcinoma with resection of the proximal humerus and implantation of a cemented modular megaprosthesis. This case highlights that the primary lesion cannot always be detected, as it may not be visualized with current diagnostic modalities or may undergo spontaneous regression. In our case, the diagnosis of renal carcinoma with proximal humeral metastasis was established following a pathological fracture of the humerus, consistent with the presentation described by Seidou et al. [8].

The decision to proceed with surgical removal of the mass and implantation of a modular prosthesis in the shoulder was crucial to restore arm functionality and improve the patient′s quality of life.

Reconstructive surgery in primary and secondary bone tumors remains a significant challenge. The use of endoprostheses for the treatment of bone tumors limited to the humerus was first described by Salzer et al. [9] and Campanacci [10].

The primary goal of limb‐salvage surgery is to eradicate the tumor while preserving the function of the shoulder girdle. The surgical plan typically consists of three steps: tumor resection, reconstruction of the bony defect, and reconstruction of the soft tissues [11].

Advanced radiological imaging and 3D reconstruction allow to simulate the level of bone resection and assist in selecting the most suitable prosthetic components (standard, custom‐made, or 3D‐printed) as well as in determining the surgical approach and whether skin flaps may be necessary [12].

Onafowokan et al. [13] reported a case of bilateral proximal humeral diaphyseal metastases from renal cancer treated with metastasis resection, trabecular metal spacer placement, and osteosynthesis using plate, screws, and cerclage wires. The patient showed good clinical and radiographic outcomes at the 2‐year follow‐up.

In our case, no attempt at osteosynthesis would have been feasible, as the humeral articular surface was entirely compromised by the neoplastic lesion.

In cases of metaphyseal metastases of the proximal humerus, the choice between osteosynthesis and megaprosthesis remains a topic of debate. A recent retrospective analysis compared outcomes of patients with proximal humeral fractures treated either with osteosynthesis (nailing) or joint replacement; the osteosynthesis group demonstrated better long‐term functional outcomes, whereas the joint replacement group showed superior local oncological control, albeit with reduced functional results [14].

Hemiarthroplasty and reverse shoulder arthroplasty have shown similar safety and complication rates following Malawer Type 1 proximal humerus resection; however, shoulder range of motion was significantly higher in the reverse shoulder arthroplasty group [15].

A 2023 meta‐analysis found that reverse shoulder arthroplasty after proximal humeral tumor resection results in an acceptable range of shoulder motion and provides good functional outcomes for daily activities (mean Constant–Murlay score of 63 points at the 2‐year follow‐up) [16].

In our case, the decision to implant a modular megaprosthesis with an EAS head rather than a reverse shoulder prosthesis was driven by several key considerations.

In the setting of advanced metastatic disease, treatment goals were tailored to the patient′s general health status, functional requirements, and projected life expectancy. The primary aim was to optimize quality of life by achieving a painless and functional limb capable of supporting ADL, while minimizing the risk of implant‐related and perioperative complications. Consequently, a less demanding reconstructive option was selected, accepting the potential trade‐off of reduced functional performance compared with reverse shoulder arthroplasty, which may have provided greater active shoulder motion.

The tumor extended in close proximity to the glenohumeral joint, with possible articular and glenoid involvement and a consequent risk of failure of a glenoid implant.

The quality of the glenoid bone stock, as assessed through preoperative imaging studies and confirmed intraoperatively, was considered inadequate to provide reliable long‐term fixation and stability of a reverse shoulder prosthesis.

Indeed, glenoid insufficiency is a well‐recognized limitation for reverse shoulder arthroplasty in oncologic reconstructions, especially following radiotherapy, which increases the risk of glenosphere loosening or mobilization [17].

This concern is supported by literature showing that although reverse shoulder megaprostheses provide acceptable functional outcomes in terms of range of motion and daily activities [16], they are not without complications, particularly when scapular bone stock is poor.

Reverse prostheses rely heavily on the stability provided by the glenosphere, which may be jeopardized in patients undergoing radiotherapy or presenting with bone loss or deformities [17].

Moreover, although functional outcomes such as shoulder flexion and abduction after reverse shoulder megaprosthesis have been reported around 105° [16], the use of an EAS prosthesis (cuff tear arthropathy [CTA] type) allowed us to avoid the additional risks related to glenoid baseplate fixation failure and to preserve what remained of the native glenoid [18]. This choice is further supported by literature emphasizing the difficulty of glenoid fixation in patients with metastatic disease or postradiotherapy alterations [19].

Therefore, based on oncologic, anatomic, and functional considerations and the need to minimize surgical and postoperative risks, the use of a CTA prosthesis was considered the most appropriate and safest option for this patient.

## 6. Conclusion

This article has outlined the complexity of selecting the most appropriate treatment strategy in the management of proximal humeral fractures secondary to metastatic CCRC.

In this case report, we opted for the use of EAS modular megaprosthesis after a comprehensive clinical assessment, reporting acceptable outcomes.

## 7. Study Limitations

This study has several limitations: single case report with retrospective nature, lack of comparative treatment groups, limited data regarding oncological treatment and treatment response, and incomplete final clinical and radiographic follow‐up. The center wherein the intervention has been performed is not a referral center for oncologic orthopedics pathologies.

The reported results should be interpreted in light of the acknowledged limitations; further studies and larger case series are warranted to validate and contextualize the conclusions drawn.

## Funding

No funding was received for this manuscript.

## Ethics Statement

This study was conducted in accordance with the principles embodied in the Declaration of Helsinki and local statutory requirements. The patient was informed about the study procedures and has signed an informed consent that authorizes publication. Ethics committee approval was not required for this case report according to local regulations. All identifying information has been anonymized to preserve patient confidentiality.

## Conflicts of Interest

The authors declare no conflicts of interest.

## Supporting information


**Supporting Information** Additional supporting information can be found online in the Supporting Information section. File S1: CARE checklist. Checklist used to ensure that the case report complies with the CARE (CAse REport) reporting guidelines.

## Data Availability

The data that support the findings of this study are available from the corresponding author upon reasonable request.
